# Seroprevalence and Molecular Detection of Bovine Viral Diarrhea Virus in Selected Dairy Farms in Southwest Ethiopia

**DOI:** 10.1155/vmi/5266912

**Published:** 2026-01-27

**Authors:** Meseret Mohammed Seid, Bruk Abraha Fitwi, Asamenew Tesfaye Melkamsew

**Affiliations:** ^1^ Department of Microbiology, Animal Health Institute, Sebeta, Oromia, Ethiopia, ahi.org; ^2^ College of Veterinary Medicine, Haramaya University, Haramaya, Oromia, Ethiopia, haramaya.edu.et

**Keywords:** antigen detection, bovine viral diarrhea, dairy farms, persistent infection, seroprevalence, Southwest Ethiopia

## Abstract

Bovine viral diarrhea (BVD) is a significant economic concern due to the risk of persistent infection and complex epidemiology in cattle‐producing countries, including Ethiopia. This study aimed to identify the circulating BVD virus type in dairy cattle, investigate seroprevalence, and assess associated risk factors in Jimma town, Oromia regional state, Southwestern Ethiopia. A cross‐sectional study was conducted from November 2023 to April 2024, involving 48 randomly selected dairy farms out of 130 registered ones. In total, 383 serum samples from BVD virus (BVDV) nonvaccinated animals were tested for BVD virus antibodies and antigens using a competitive enzyme‐linked immunosorbent assay (ELISA) kit (ID Screen BVD p80 antibody). Additionally, a one‐step reverse transcription polymerase chain reaction (RT‐PCR) was used to detect the viral genome in pooled swab samples. Analytical statistics, including chi‐square and multivariable logistic regression, were employed for data analysis using SPSS, Version 26. The study revealed that 72 animals (18.8%) and 20 farms (41.7%) tested positive for BVDV antibodies. All the tested samples were negative for BVDV antigen and viral genome. Age, history of respiratory problems, breeding system, and housing system were statistically associated with seroprevalence (*p* < 0.05). At the farm level, only the production system showed a significant association (*p* < 0.05). Adult animals had 2.2 times the odds of being seropositive (OR = 2.2; *p* = 0.02). Animals with respiratory issues and those housed in head‐to‐tail arrangements had 2.7 (*p* = 0.205) and 4.8 (*p* = 0.021) times the odds of being seropositive, respectively. In conclusion, a substantial proportion of dairy cattle in the study area are exposed to BVD virus. However, no evidence of persistent infection was found among the dairy farms. Effective management strategies are crucial, including vaccination, biosecurity measures, and housing management.

## 1. Introduction

One of the most important viral diseases affecting cattle globally is bovine viral diarrhea (BVD) [1, 2]. The virus is a single linear positive‐stranded RNA virus belonging to the genus Pestiviruses in the family *Flaviviridae* [2]. The two separate species or genotypes of BVDV are Type I and Type II [3], which can be distinguished from other Pestiviruses by targeting the E2 protein using genomic and immunologic assays [4]. Based on the growth character, BVDV can be further categorized into cytopathic (CP) and non‐CP (NCP) subtypes [5].

Acute or transitory infection and persistent infection (PI) are the two disease progresses linked to BVDV. Acute infection happens due to a postnatal infection in an immunocompetent host, while PI only occurs when the NCP type of BVDV establishes infection in the uterus of a dam, followed by the subsequent infection of a fetus before immunocompetency, in a phenomenon called immunotolerance [2, 6]. It is responsible for morbidity, mortality, the increased rate of early culling, decreased milk output, and impaired reproductive performance [7, 8].

The most common way of naturally occurring infection with BVDV is direct contact through the oronasal route, because BVDV persistence in the environment is limited to a few days, which signifies the importance of acutely infected or PI animals in BVDV epidemiology. More importantly, persistently infected (PI) animals play a key role in the spreading of BVDV, because they cannot acquire antibodies against BVDV and continuously excrete large amounts of the virus undetectably [9]. Detection of virus‐specific antibody using different serological tests, such as virus neutralization test and enzyme‐linked immunosorbent assay (ELISA), is used for the indirect detection of the virus [10]. BVDV antigen‐ELISA and reverse transcription polymerase chain reaction (RT‐PCR) are the two most reliable and sensitive methods for detecting PI animals, with antigen‐ELISA being the most cost‐effective for testing large numbers of animals [9].

In the global cattle population, the seroprevalence of BVD ranges from 46.2% to 48.7% at the animal level and 66% to 67% at the herd level [11]. In Ethiopia, animal‐level prevalence ranges from 9.59% to 51.7% [12–16], while a herd prevalence was reported to be 50% [16]. The major strategies for prevention and control of BVDV include the identification and elimination of PI animals, enhanced immunity through vaccination, and implementation of biosecurity measures. Despite the advances in the detection of PI animals [17], research related to the BVDV in Ethiopia has focused primarily on adult cattle, with limited attention given to the identification of PI calves. This study aimed to investigate the seroprevalence of BVDV from selected dairy farms in Jimma town, identify persistently infected calves using RT‐PCR and antigen detection ELISA, and assess the potential risk factors associated with the seroprevalence of BVDV in the study animals.

## 2. Materials and Methods

### 2.1. Study Area Description

The study was conducted on selected dairy farms in Jimma town, located in the Jimma Zone of the southwest Oromia region. Jimma town is located at a latitude and longitude, from 7°40′00″ up to 7°42′00″N, and from 42°47′30″ up to 42°53′30″E, respectively, and an elevation of 1780 m above sea level (Figure 1). The mean annual rainfall of the town is 1530 mm, which comes from the long and short rain seasons. The mean annual minimum and maximum temperatures are 14.4°C and 26.7°C, respectively, and the relative humidity is 61.3% [18]. Jima town livestock population is estimated at 58,765 cattle, 27,853 sheep, 13,877 goats, 11,073 equines, and 99,533 poultry. In the town, there were an estimated 6000 cross‐bred dairy animals [19].

**Figure FIGURE 1:**
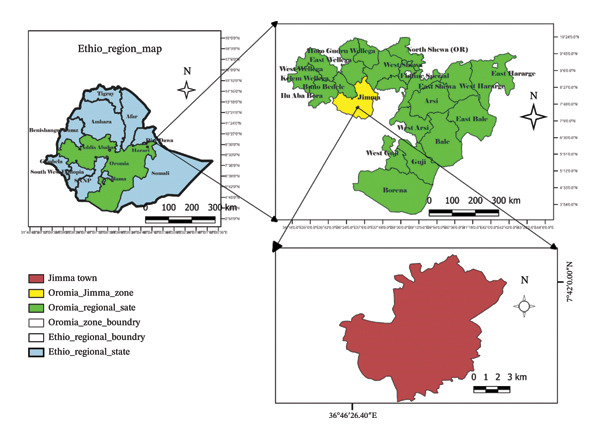
A map of the study area. *Source:* ArcGIS, USA California, 2023.

### 2.2. Study Population

For the seroprevalence study, the animals considered include those > 6 months of age, both sexes, and Holstein Friesian cross‐breed cattle managed in all production systems (intensive, semi‐intensive, and extensive) and scales of production (small, medium, and large scale) with or without clinical signs of the disease. The detection of persistently infected calves was based on viral antigen detection (antigen‐capture ELISA and RT‐PCR), and only calves aged 3 to 6 months were considered, given their lower risk of acute, transient infection compared to older animals (an indication of waned immunity). All the cattle included in the study group had never been immunized against BVDV because there is no BVDV vaccination program in Ethiopia. The study animals were managed under intensive and mixed farming systems of the urban area (i.e., Jimma town). Hand milking was practiced in all the study farms.

### 2.3. Study Design and Sampling

The cross‐sectional study was conducted from November 2023 to April 2024. Relevant individual animal and farm‐level information was collected using a semistructured questionnaire. The sample size for the seroprevalence was calculated according to Thrusfield et al. [20] formula. Thus, based on the estimated prevalence of 51.9% [15] from Jimma town, assuming simple random sampling, the sample size was expressed as follows:

(1) n=z2Pexp1−Pexpd2.

Accordingly, with a 95% confidence level and 5% desired precision (*d* ^2^), a total of 383 animals were randomly selected for serum sample collection. Out of the 130 registered dairy farms in Jimma town, a total of 48 dairy farms were selected randomly. Individual dairy cattle were selected after listing based on their identification system (i.e., ear tags and names given by the owner). Thus, a lottery system was applied to ensure that approximately 50% of animals on the farm were included in serum sample collection based on a probability sampling approach.

### 2.4. Sample and Data Collection

From individual animals, 8 mL of whole blood samples in adult cattle and 5 mL of whole blood in young were collected by puncturing the jugular vein using a 21‐gauge needle with a needle holder and a plain vacutainer tube (Henso vacuum tube: Zhejiang Kangshi Medical Devices Co. Ltd., Hangzhou, China). Then, it was kept at room temperature in a slant position overnight, and the separation of serum samples was made using appropriately leveled cryovial tubes. All the serum samples were transported using a cool box and stored at −20°C in Jimma University, School of Veterinary Medicine, Veterinary Microbiology and Public Health Laboratory, Jimma, Ethiopia. The nasal orifice was properly cleaned using clean and sterile cotton before sampling, and swab samples were collected using clean, sterile cotton swabs inserted into the nasal cavity to touch the back of the nasal septum from both nostrils [21]. Swabs were transferred into a properly labeled, clean, sterile cryovial tube containing a viral transport medium (300 μL VTM) and were kept in liquid until shipped to the Animal Health Institute (Sebeta, Ethiopia). Upon arrival, swab samples were stored at −80°C, while serum samples were kept at −20°C until the laboratory analysis was conducted.

Relevant animal‐ and farm‐level information was collected using a semistructured questionnaire. Thus, information collected included farm size, animal housing system, age, breed, body condition status, means of reproduction (natural or artificial insemination [AI]), the origin of replacement stock (external, same farm, mixed), calf mortality history, history of respiratory problem, and persistent diarrhea. Age and farm size categorization were according to the national livestock data standard [22], considering the dairy farming system. Thus, young animals were those less than 3 years, and adults were those 3 years and above. The body condition was categorized as average (medium) and heavy (good) according to the Department for Environment, Food and Rural Affairs [23].

### 2.5. Antibody Detection by ELISA

ID Screen BVD p80 Antibody Competition ELISA, developed by Louis Pasteur Institute in Grabels, France, was used to detect antibodies against BVDV. Briefly, the frozen serum samples were thawed at room temperature for 15–20 min. A diluted 50 μL serum sample and 20 μL controls are added to microplates coated with p80 protein. The test samples were incubated in the coated wells at 37°C for 2 h, allowing p80 protein‐specific antibodies to form immune complexes. After washing unbound antibodies, an enzyme‐conjugated anti‐p80 protein antibody (HRP) was added and incubated at 37°C for 1 h. In the presence of the p80 protein–antibody complex, the conjugate was blocked from binding to corresponding epitopes on the microplates. Conversely, in the absence of the p80 protein–antibody complex, the conjugate binds to its corresponding epitopes. Unbound conjugate was washed away, and the enzyme substrate (TMB) was added. The enzyme oxidizes the substrate, resulting in a blue compound that turns yellow after adding a stop solution. The subsequent color development is inversely proportional to the amount of anti‐p80 protein antibodies in the test sample. The test was valid when the mean optical density of the negative control (ODNC) to optical density of the positive control (ODPC) is > 0.7, and the ratio of the positive control to the negative control (ODPC/ODNC) < 0.3 according to the manufacturer’s instruction (iD.vet BVDC Ver. 0117 EN protocol 1/2).

### 2.6. Antigen Detection by ELISA

ID Screen BVD p80 Antigen Capture ELISA, developed by the Louis Pasteur Institute (Grabels, France), was used according to the manufacturer’s instructions. Frozen serum samples were thawed at room temperature for 15–20 min. A diluted serum sample (50 μL) and 20 μL of positive and negative controls were added to microplates coated with p80 protein. In the wells, the BVDV antigen was captured by specific antibodies. After washing, a ready‐made conjugate (anti‐BVDV‐p80, 100 μL HRP) was added, forming an antibody–antigen–antibody–HRP complex. Test samples were incubated in the coated wells at 37°C for 2 h. Unbound conjugate was washed away three times with 300 μL wash solution, to prevent nonspecific binding and background signal and ensure that the subsequent substrate reaction reflects only antigen‐specific complexes. Then, 100 μL of substrate/chromogen solution was added, which subsequently generates a blue color. Upon the addition of 100 μL of stop solution, a yellow color developed. Absorbance was measured using a spectrophotometer (BioTek ELx 8081U, USA) at 450 nm wavelength. The test was considered valid when the mean ODNC to the ODPC > 0.5, and the ratio of ODPC to ODNC > 3.

### 2.7. Viral RNA Detection by RT‐PCR

NucleoSpin RNA Virus Mini kit (Macherey‐Nagel, Germany) was used to isolate total RNA according to the manufacturer’s instructions (Cat. No. 740962.20). SuperScript IV One‐Step RT‐PCR (Invitrogen, USA) was used during the PCR analysis. The primers used to amplify 5′UTR were adapted from Gong et al. [24]. The PCR amplification was conducted in a total volume of 50 μL containing 25 μL of 2x platinum SuperFi RT‐PCR master mix, 2.5 μL of forward primer, 2.5 μL of reverse primer, 0.5 μL of SuperScript IV RT mix, 2 μL of template RNA, and 17.5 μL of nuclease‐free water. The PCR cycling involved reverse transcription at 50°C for 10 min and RT inactivation/initial denaturation at 98°C for 2 min, followed by 40 cycles at 98°C for 10s, 57°C for 10s, and 72°C for 1 min, with a final elongation step at 72°C for 5 min. The amplicons were subjected to 1.5% agarose gel electrophoresis and visualized following ethidium bromide staining.

### 2.8. Defining PI Animals

An animal is defined as persistently infected if it is viremic (virus‐positive) and antibody‐negative (seronegative) [2]. Thus, the test results of antigen‐capture ELISA and RT‐PCR were considered for virus positivity. For the molecular detection, samples were pooled randomly to minimize the economic costs of testing and with the assumption that pools will detect the single PI animal by testing individually to identify the respective BVDV positive animal from the positive pools [9, 25, 26].

### 2.9. Data Analysis

Data were transferred to IBM SPSS software, Version 26 (IBM, Chicago, USA) for analysis. Descriptive statistics such as frequency and percentages were used to describe the seroprevalence of BVDV. The association of animal factors and farm factors was analyzed using the *χ* ^2^ test, followed by multivariable logistic regression analysis to measure the magnitude of association between explanatory variables and seroprevalence of BVDV at the animal level. Thus, the odds ratio (OR) with 95% confidence intervals (CIs) was computed based on the assumptions of Thrusfield et al. [20]. The criteria used for multivariable logistic regression analysis were the association of explanatory variables with seroprevalence and multicollinearity test [27, 28]. Upon univariable logistic regression analysis, sex, body condition, the introduction of new animal, and persistent diarrhea showed a *p* value greater than (equals to) 0.25 and were excluded from the final model of the multivariable regression model [29]. A value of *p*  <  0.05 was considered significant.

### 2.10. Ethical Approval

This study was approved by the ethics review committee of the National Animal Health Diagnostic and Investigation Center (NAHDIC), Ministry of Agriculture, with Certificate Number ARSERC/EC/022/26/08/2022. The study was conducted following the national and international recommendations on humane handling of animals.

## 3. Results

### 3.1. Seroprevalence of BVDV and Associated Risk Factors

In the current study, 383 blood samples were analyzed using indirect ELISA. Out of these, 72 (18.8%; 95% CI = 14.9–22.7) were found positive for BVDV antibodies. The animal‐level prevalence of BVDV was assessed based on risk factors that were considered to have an association with the occurrence of BVDV (Table 1). Based on the chi‐square analysis, sex, farm size, body condition, and persistent diarrhea did not show a significant association (*p* > 0.05) with BVDV seroprevalence. However, the prevalence was relatively higher in females (19.4%) than in males (12.5%). Moreover, animals in small farm size (21.8%) had a relatively higher prevalence than those in large farms (16.5%). On the other hand, the prevalence was significantly (*p* < 0.05) higher in adult animals (26.2%) than in the younger category (9.5%). The odds of being positive for BVDV antibodies were 2.2 times in adults compared to young animals (OR = 2.2; 95% CI = 1.1–4.4), and the variation was significant (*p* = 0.02). Animals with respiratory symptoms had a significantly higher (*p* < 0.001) seroprevalence (21.1%) than those without symptoms (3.8%), and in animals with respiratory symptoms, the odds of being seropositive were 2.7 (OR = 2.7; 95% CI = 0.6–13.0; *p* value = 0.205).

**Table TABLE 1:** Individual animal‐level prevalence using chi‐square and multivariable logistic regression analysis.

Variable	Category	*N*	No. (%) positive	95% CI	χ^2^ analysis	Regression analysis			
					χ^2^ (*p* value)	COR (95% CI)	*p* value	AOR (95% CI)	*p* value
Sex	Male	32	4 (12.5)	3.5–28.9	0.9 (0.48)	Ref		NA	NA
	Female	351	68 (19.4)	15.3–23.9		1.6 (1.7–4.9)	0.35	NA	NA

Body condition	Medium	162	28 (17.3)	11.8–24.0	0.4 (0.59)	Ref		NA	NA
	Good	221	44 (19.9)	14.9–25.8		1.2 (1.4–2.0)	0.52	NA	NA

Persistent diarrhea	Present	124	21 (16.9)	10.8–24.7	0.4 (0.58)	Ref		NA	NA
	Absent	259	51 (19.7)	15.0–25.0		1.2 (1.5–2.1)	0.52	NA	NA

Age	Young	169	16 (9.5)	5.5–14.9	17.3 (< 0.001)	Ref		Ref	
	Adult	214	56 (26.2)	20.4–32.6		3.4 (0.5–6.2)	< 0.001	2.2 (1.1–4.4)	0.02

New animal	Absent	142	26 (18.3)	12.3–25.7	0.03 (0.9)	Ref		NA	NA
	Present	241	46 (19.1)	14.3–24.6		1.1 (1.6–1.8)	0.85	NA	NA

Respiratory symptom	Absent	52	2 (3.8)	0.4–13.2	8.8 (< 0.001)	Ref		Ref	
	Present	331	70 (21.1)	16.9–25.9		6.7 (0.6–28.6)	0.009	2.7 (0.6–13.0)	0.205

Herd size	Large	218	36 (16.5)	11.8–22.1	1.7 (0.19)	Ref		Ref	
	Small	165	36 (21.8)	15.8–28.9		1.4 (0.8–2.4)	0.18	1.1 (2.2–2.4)	0.934

Production system	Mixed	40	0 (0.0)	0–13.2	10.34 (< 0.001)	—	—	—	—
	Intensive	343	72 (21.0)	16.8–25.7		—	—	—	—

Breeding	Both	74	7 (9.5)	3.9–18.5	5.2 (0.02)	Ref		Ref	
	AI	309	65 (21.0)	16.6–26.0		2.6 (0.9–5.8)	0.03	1.9 (0.7–4.9)	0.18

Animal housing	Single row	50	3 (6.0)	1.3–16.5	13.01 (< 0.001)	Ref		Ref	
	TT	38	3 (7.9)	1.6–21.4		1.3 (3.9–7.0)	0.74	1.3 (4.7–8.5)	0.76
	HT	91	25 (27.5)	18.6–37.8		5.9 (0.6–20.8)	0.005	4.8 (1.3–18.4)	0.021
	HH	204	41 (20.1)	14.8–26.3		3.9 (0.9–13.3)	0.027	2.9 (0.7–12.1)	0.13

*Note: N* = Number of examined animals; NA = Not applicable (i.e., removed from the final model).

Abbreviations: AI, Artificial insemination; AOR, Adjusted odds ratio; COR, Crude odds ratio; HH, Head‐to‐head; HT, Head‐to‐tail; TT, Tail‐to‐tail.

Animals that are found in farms practicing AI for breeding had significantly (*p* < 0.05) higher (21.0%) BVDV seroprevalence than those found in farms using both AI and natural matting (9.5%), with the odds of being positive in the former group to be 1.9 (OR = 1.9; 95% CI = 0.7–4.9; *p* value = 0.18). Seropositivity to BVDV showed a significant association with the arrangement of animals in the farm (Figure 2). Among the animal housing, head‐to‐tail had the highest prevalence (27.5%), followed by head‐to‐head (20.1%), tail‐to‐tail (7.9%), and single row (6%). As compared to animals managed under the one‐row system, animals kept under head‐to‐tail and head‐to‐head management systems had OR values of 4.8 (OR = 4.8; 95% CI = 1.3–18.4 CI; *p* value = 0.02) and 2.9 (OR = 2.9; 95% CI = 0.7–12.1; *p* value = 0.13), respectively.

Figure FIGURE 2Different arrangements of dairy cattle in Jimma town. Head to head (a) and tail to tail (b).

**Figure:**
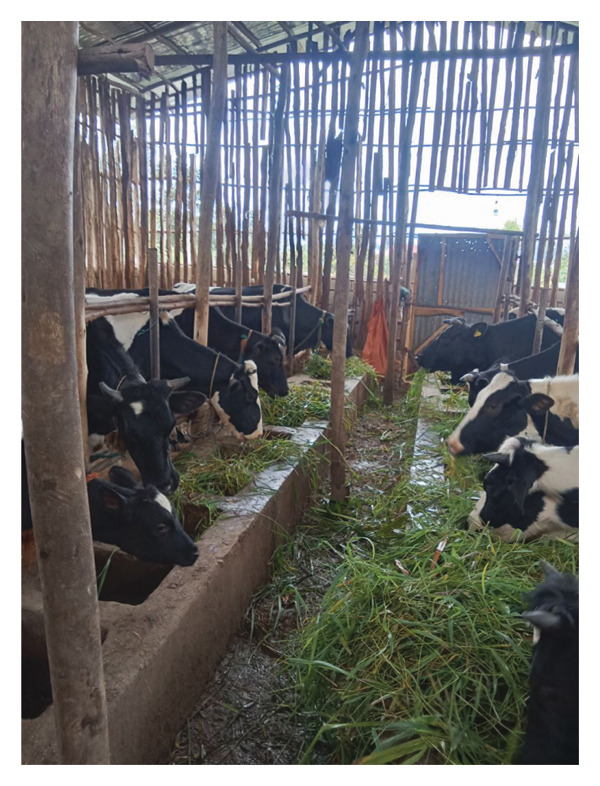
(a)

**Figure:**
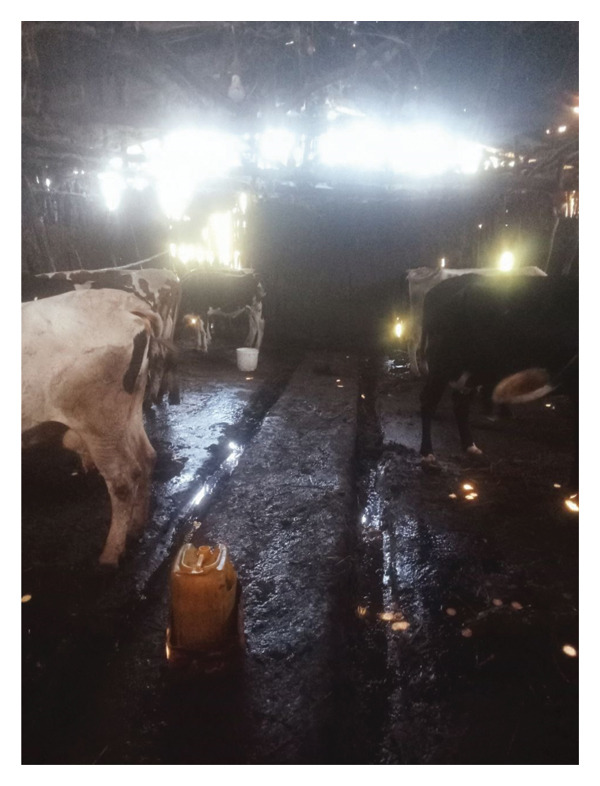
(b)

The finding on risk factors associated with the prevalence of BVDV at the farm level is presented in Table 2. The production system showed a significant association (*p* < 0.05) in that 46.5% of the intensive production systems had at least one seropositive animal, while in the mixed farming systems, no animal (0.0%) was found to be positive. When considering the herd size, small herds had a higher prevalence of BVDV (57.9%) compared to large herds (31.0%), but with no significant association (*p* > 0.05).

**Table TABLE 2:** Farm‐level prevalence analysis using chi‐square and multivariable logistic regression analysis.

Variable	Categories	No. of farms examined	No. (%) positive	95% CI	*X* ^2^ value	*p* value
Herd size	Small	19	11 (57.9)	33.4–79.7	3.41	0.06
	Large	29	9 (31.0)	15.3–50.8		

Production system	Intensive	43	20 (46.5)	31.2–62.3	3.9	0.04
	Mixed	5	0 (0)	0.0–71.6		

Breeding	AI	40	18 (45)	29.3–61.5	1.1	0.44
	Both/Natural	8	2 (25)	3.1–65.0		

Animal housing	Head‐to‐head	19	10 (52.6)	28.9–75.5	4.5	0.2
	Head‐to‐tail	14	7 (50)	23.0–76.9		
	Tail‐to‐tail	3	1 (33.3)	0.8–90.6		
	Single row	12	2 (16.7)	2–48.4		

New animals	Present	27	11 (40.7)	22.4–61.2	0.02	1.0
	Absent	21	9 (42.9)	21.8–65.9		

Calf mortality	Present	33	14 (42.4)	25.5–60.8	0.02	1.0
	Absent	15	6 (40.0)	16.3–67.7		

Persistent diarrhea	Present	16	5 (31.2)	11.0–58.7	1.1	0.30
	Absent	32	15 (46.9)	29.1–65.3		

Respiratory symptom	Present	37	18 (48.6)	31.9–65.6	3.2	0.07
	Absent	11	2 (18.2)	2.2–51.8		

Abbreviation: AI, Artificial insemination.

### 3.2. Detection of Persistently Infected Calves

In the current study, 150 nasal swab samples were collected from calves to detect persistently infected (PI) calves and tested using RT‐PCR. The test was conducted after pooling into 12 groups. However, during RT‐PCR amplification, no bands were detected (Figure 3). Moreover, the serum samples collected from the same samples tested negative for the antigen detection ELISA.

**Figure FIGURE 3:**
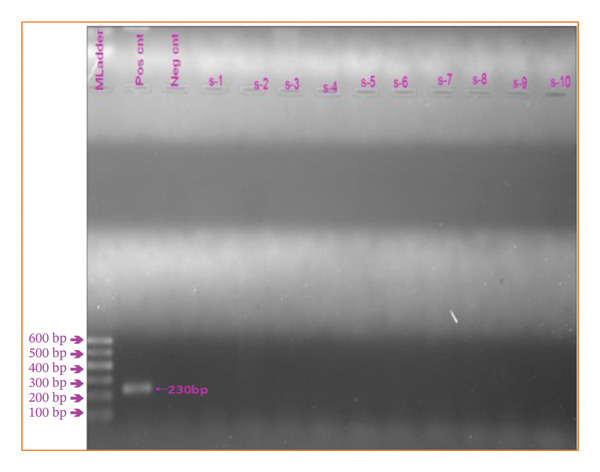
Gel electrophoresis for the 5′UTR regions of BVDV (230‐bp amplified product). Lane *M* = 100‐bp DNA ladder; Pos cnt = positive control (NADL strain); Neg cnt = negative control; S‐1 to S‐10 = coded pooled samples indicating no presence of expected bands.

## 4. Discussion

A significant pathogen in cattle that causes respiratory illnesses, enteritis, and immune system dysfunction is BVDV. When a vulnerable dam contracts the NCP strain of the BVDV and goes viremic early in gestation (between 45 and 125 days of cow pregnancy), the fetus gets infected through the placenta. If the infection has occurred before the immune system is mature, the calves become persistently infected with the BVDV. Persistent infection in calves leads to birth defects, abortions, poor growth, susceptibility to diseases, and death at an early age [30]. However, the threat of persistent infection is that PI calves may appear healthy and grow well, but become clinically silent life‐long shedders of the virus among the animal population [31]. The methods widely used for BVDV detection include virus isolation (i.e., the gold standard test), immunohistochemical, PCR, and ELISA (both direct and indirect). However, their application depends on the nature of the sample and the objective of detection. RT‐PCR assay has been developed for the detection of BVDV in a wide variety of clinical samples, including blood [32], serum [33], and nasal swabs [34]. In this study, an attempt was made to determine the seroprevalence and detect the presence of BVDV using ELISA and RT‐PCR from serum samples and nasal swabs, respectively. As the direct culture of all collected samples is costly, we first screened the samples (i.e., pooled) using an RT‐PCR test. However, the RT‐PCR result was negative, and we could not proceed with the virus isolation. The current study’s detection of PI was based on a combination of antibody, antigen, and viral genome, which represents the first of its kind in the country.

The current study has shown that animal‐level seroprevalence and farm‐level seroprevalence were 18.8% (95% CI = 15.0–23.1) and 41.7% (95% CI = 27.6%‐56.7%), respectively, which agrees with the study of Aragaw et al. [16], who showed animal‐level prevalence of 20.9% and farm‐level prevalence of 50% in different parts of the country. The current study’s farm‐level prevalence partly agrees with the report of Asmare et al. [13], who showed a prevalence of 38% (in southern Ethiopia) and 19% (in central Ethiopia). Likewise, Callaby et al. [35] reported a prevalence of 19.8% in Kenya. However, it was higher compared to the previous report by Nigussie et al. [12], who reported an animal‐level prevalence of 9.59% in the Jimma Zone and 6.11%–16.6% in different agro‐ecological zones of the country.

On the other hand, the animal‐level prevalence is lower than previous prevalence reports such as 51.9% in Jimma town [15]; 32.6% (ranges from 29.1% to 38.5%) in different parts of Ethiopia [14]; 48.8% in Iran [36]; 51.1% in Bangladesh [37]; 54.3% in Brazil [38]; 36% in Colombia [39]; 40% in Egypt [40]; and 27% in Ecuador [41]. In Jimma town, Tadesse et al. [15] reported a higher herd level prevalence (95.6%). Likewise, Aragaw et al. [14] reported an average prevalence of 68.9%, which ranges from 64.2% to 100% at different milk sheds in the country.

The divergence between the findings of the current study and those reported earlier can be attributed to sample size, which may affect the statistical power necessary to discern significant associations. In other words, larger‐scale studies tend to furnish more robust and dependable results. Moreover, disparities in study design and measurement methods can contribute significantly [20]. Other factors contributing to disparities in prevalence reports include the timing of BVDV detection (i.e., whether during acute or chronic phases), the study animals’ genetic diversity, and management practices [11]. Moreover, it was previously reported that the geographical variation further compounds this complexity, as BVDV prevalence fluctuates across regions and climates [42].

The current study revealed that BVDV seroprevalence was significantly (*p* < 0.05) higher in adult animals, which is consistent with the findings of Daves et al. [43] who reported 36.7% in adults and 15.2% in young animals. Likewise, Aragaw et al. [14] and Tadesse et al. [15] reported a significantly higher prevalence in adult animals. Generally, adult animals exposed to BVDV mount strong immune responses as compared to young animals [44]. Due to frequent exposure to different animals, environments, and contaminated surfaces, adults have more time to encounter various pathogens, including BVD [45–47]. Young calves are often kept separate from other age groups, reducing their exposure. Adults, especially those in mixed‐age herds, face higher risks due to the less stringent biosecurity practices [48]. Adult cattle may experience stress due to factors such as transportation, changes in management, or social dynamics within the herd. Stress weakens the immune system, making them more susceptible to infections by BVDV [49]. Adults are more likely to encounter PI animals over time, increasing their risk of exposure [50]. Other studies have explained that a lower seroprevalence in calves could also be due to some of the calves investigated being PI animals, which are known to be immunotolerant to the virus and do not produce antibodies [51, 52]. However, this is unlikely in the current study, because antigen detection (using antigen‐capture ELISA and PCR) did not reveal the presence of a circulating virus.

The current finding of a significantly higher (*p* < 0.05) prevalence of BVDV in animals with a history of respiratory problems agrees with previous literature and reports. For instance, BVDV‐neutralizing antibodies were commonly reported in nonvaccinated animals with a clinical history of respiratory signs [53, 54]. This could be due to concurrent infections with other respiratory pathogens such as bovine respiratory syncytial virus [55] and bovine coronavirus infections [56], which ultimately play an important role in bovine respiratory syndromes.

The currently reported significantly higher (*p* < 0.05) prevalence in animals managed at farms practicing AI (21.0%) for breeding than those used both AI and natural (9.5%) disagrees with the report of Aragaw et al. [16], who reported a relatively higher (*p* > 0.05) prevalence in farms using natural (24.1%), followed by AI (21.4%) and both (19.8%). However, the current finding of a relatively higher prevalence of BVDV in farms practicing AI (45%) than in mixed (25%) agrees with the finding of Tadesse et al. [15] in the same study area, in that they reported a higher prevalence in farms using AI (97.5%) than those used natural mating (80%) and the difference is significant (*p* < 0.05). Despite these conflicting reports, studies conducted by Meyling and Jensen [57] and Kirkland et al. [58] demonstrated that BVDV can be transmitted in cattle with semen having high virus titer during artificial insemination.

Concerning the sex of animals, the current report of relatively higher (*p* > 0.05) prevalence in females (19.4%) than males (12.5%) disagrees with the reports of Tadesse et al. [15] from Jimma Town, who showed significantly higher (*p* < 0.05) prevalence in females than males. Moreover, it is not in agreement with the findings of Daves et al. [43], who reported a significantly higher prevalence (*p* < 0.05) in females (35.5%) than males (16.3%). This variation could be due to the relatively small samples used in the current and previous studies, which ultimately affects the power of statistical models. For instance, in their study, Tadesse et al. [15] involved 402 females and 18 males. Likewise, Daves et al. [43] involved 358 females and 49 males. Generally, the sex‐based variation could be related to the practice of early removal of male calves in dairy farms for different purposes [59].

Contrary to the current finding, Aragaw et al. [14] and Aragaw et al. [16] showed that the prevalence was higher in large‐sized farms than in small. Concerning herd size, it is challenging to directly compare because different researchers use different criteria for classifying. For instance, Tadesse et al. [15] classified > 5 animals as large, while some used < 10 cows to be small. These disparities are faced by the challenges of animal number and the group of animals to be considered, in that some consider only cows (excluding other categories).

In the current study, the observed high prevalence of BVDV in intensively managed dairy cattle could be due to confinement, which leads to the high risk of transmission through respiratory secretions, and shared resources, such as feeding troughs, waterers, and handling equipment [16, 60]. In intensive farming systems, sick animals may not be easily isolated from healthy ones and can spread BVDV silently (unknowingly) to susceptible animals [61]. Moreover, stress is common in confined animals, and this can weaken the immune system, which in turn makes them more susceptible to infections like BVD [62].

The current study reported that as compared to the animals kept under the single‐row system, those managed under head‐to‐tail and head‐to‐head systems are 4.8 times and 2.9 times more likely to be positive for BVDV, respectively, which partly agrees with the study by Asmare et al. [63], who reported that keeping animals under head‐to‐head and one‐row systems had 3.6 and 3.0 times odds of being seropositive as compared to animals kept under tail‐to‐tail systems, respectively. Moreover, Aragaw et al. [16] reported that the odds were high in head‐to‐head arrangements (OR = 1.7), but contrasting regarding the single‐row system, which was reported as the next higher risk (OR = 1.5) as compared to the other systems. In a single‐row arrangement, animals have limited direct contact with each other compared to more densely packed arrangements. Reduced contact minimizes the chances of virus transmission through nose‐to‐nose or mouth‐to‐mouth contact with infected animals. Single‐row arrangements often provide more space between animals, allowing for better isolation [64, 65]. Moreover, single‐row arrangements may have fewer PI animals, leading to a lower overall infection risk in the herd [66].

Animals in a head‐to‐tail arrangement might have reduced face‐to‐face contact, but it does not prevent other forms of physical contact (e.g., tail‐to‐nose). Moreover, the BVDV can spread through contaminated surfaces (such as feed bunks, water troughs, and fences). Thus, animals in a head‐to‐tail system may still encounter these contaminated surfaces, leading to potential transmission of BVDV [67]. Likewise, in a head‐to‐head arrangement, animals are positioned nearby, often facing each other. This physical closeness facilitates the transmission of infectious agents, including BVD. BVD is primarily spread through direct contact with bodily fluids (such as saliva, nasal discharge, urine, and feces) from infected animals. It is usual practice that animals managed head‐to‐head often share common feed troughs, waterers, and other resources [68].

The current study’s finding on the association of the introduction of new animals with BVDV is in line with the findings of Tadesse et al. [15] from Jimma, who reported a higher prevalence in farms exposed to new animals, which is contrary to the current finding. Generally, risk factor assessment is affected by the presence of uncontrolled variables (commonly referred to as confounders), which introduce additional layers of complexity and exert an influence on associations [20]. In particular, it is essential to recognize that BVDV infection is a multifactorial phenomenon, implicating immunity [69], stress, and biosecurity measures [70, 71]. Also, herd dynamics, management practices, and exposure levels influence BVDV prevalence [42].

In the present study, 150 nasal swab samples of calves were pooled into 12 nasal swab pools (each pool contained 12 samples), and one nasal swab pool contained 18 swab samples. Moreover, antigen‐capture ELISA was employed to achieve antigen detection in serum from the same samples pooled for one‐step RT‐PCR. All the samples examined tested negative for RT‐PCR, which is in line with the report of Ince and Ahmet [72] from Kenya, who tested negative for antigen and BVDV‐specific RNA. From Ethiopia, Yitagesu et al. [65] used the antigen‐capture ELISA to screen ear‐notch samples for the presence of the BVDV antigen but found negative for all samples. According to Mokhtar et al. [73], the detection of the BVDV genome by PCR in pooled bovine serum, fetal fluid, and seminal fluid is possible. The authors have also noted that PI animals were detectable in pools of 200–250 seronegative samples. To avoid false negatives due to sample dilution, the maximum number of samples can be assigned to a pool of 80 based on herds [24]. Moreover, Khodakaram‐Tafti and Farjanikish [74] noted that pooling strategies to detect the BVDV in persistently infected cattle are popular options due to lower testing costs because persistently infected animals are frequently less than 1% of the animals in the herd.

The absence of antigen detection could be due to the reason that some animals may experience transient BVDV infections due to exposure to infected animals. These transient infections may not yield consistent PCR or antigen‐positive results [75]. Another possible explanation is that recent BVDV vaccination with live vaccines can contribute to transient infection. However, this is unlikely for the current study, because no‐vaccination programs are currently undergone in the study area. On the other hand, since PIs are only associated with the occurrence of circulating NCP biotypes, antigen detection tests could fail to detect BVDV [76, 77]. However, the failure to detect BVDV antigen (ELISA and PCR) might indicate a rare occurrence of PI calves, because it was previously reported that PI animals tend to consistently test positive for BVDV over time, but not in transiently infected animals [78].

However, due consideration should be given to the possibilities of varying sensitivities during RT‐PCR and antigen tests, particularly, to low viral load and sampling errors. In addition, BVDV exhibits high genomic sequence variability and different BVDV strains may evade detection by specific RT‐PCR primers or antigen‐based assays [79]. Moreover, BVDV shedding patterns vary during acute and chronic phases, which influences antigen detection using ELISA. In summary, the absence of antigen detection during BVDV studies results from a complex interplay of factors, including transient infections, diagnostic limitations, strain variability, and population‐specific dynamics [2]. Moreover, it was previously reported that BVDV field strain variability needs to be considered to improve the sensitivities of molecular diagnostic and optimize immunization protocols [80].

## 5. Conclusions

The serological investigation revealed that a considerable proportion (18.8%) of dairy cattle and farms (41.7%) had exposure to BVDV. Compared to previous reports, the prevalence varied, highlighting the complex nature of BVD epidemiology. Factors like age and respiratory issues were associated with seroconversion. Farm‐level practices, such as production systems, played a role in disease epidemiology. Breeding systems also impact BVDV prevalence. Despite testing, no circulating virus was found during the study. Even one BVDV‐infected animal can contribute to the virus’s spread within a farm and across regions. Considering BVDV’s genetic diversity, undetected cases should be considered. Therefore, it is suggested to conduct rigorous testing based on a longitudinal study design, which includes as many animals as possible. Moreover, considering the multifactorial nature of BVDV epidemiology, studies should consider the herd dynamics in conjunction with seasonal and other related factors. This study also gives a foundation to the investigation of the circulating BVDV strains in breeding centers.

## Author Contributions

Meseret Mohammed Seid, Bruk Abraha Fitwi, and Asamenew Tesfaye Melkamsew designed the study. Meseret Mohammed Seid participated in sample collection and analysis. Meseret Mohammed Seid and Bruk Abraha Fitwi performed the data analysis and drafted the manuscript.

## Funding

The authors declare that no financial support was received for the research and/or publication of this article.

## Disclosure

All authors read and approved the final manuscript.

## Ethics Statement

Ethical approval was issued by the ethical clearance committee of the National Animal Health Diagnostic and Investigation Center (NAHDIC) with Certificate Number ARSERC/EC/022/26/08/2022. Written informed consent was not obtained from the owners for the participation of their animals in this study because all the dairy farmers who were willing to be part of the study were briefed about the objective of this study. Only information about individual animals and farm characteristics was collected.

## Conflicts of Interest

The authors declare no conflicts of interest.

## Data Availability

The data that support the findings of this study are available from the corresponding author upon reasonable request.
